# Advanced Glycation End Products Enhance Macrophages Polarization into M1 Phenotype through Activating RAGE/NF-*κ*B Pathway

**DOI:** 10.1155/2015/732450

**Published:** 2015-05-31

**Authors:** Xian Jin, Tongqing Yao, Zhong'e Zhou, Jian Zhu, Song Zhang, Wei Hu, Chengxing Shen

**Affiliations:** ^1^Department of Cardiology, Xinhua Hospital, Shanghai Jiaotong University School of Medicine, 1665 Kongjiang Road, Shanghai 200092, China; ^2^Department of Cardiology, Central Hospital of Minhang District, 170 Xinsong Road, Shanghai 201199, China; ^3^Department of Cardiology, Zhongda Hospital Affiliated to Southeast University, 89 Dingjiaqiao Road, Nanjing 210009, China

## Abstract

Atherosclerotic lesions are accelerated in patients with diabetes. M1 (classically activated in contrast to M2 alternatively activated) macrophages play key roles in the progression of atherosclerosis. Since advanced glycation end products (AGEs) are major pathogenic factors and active inflammation inducers in diabetes mellitus, this study assessed the effects of AGEs on macrophage polarization. The present study showed that AGEs significantly promoted macrophages to express IL-6 and TNF-*α*. M1 macrophage markers such as iNOS and surface markers including CD11c and CD86 were significantly upregulated while M2 macrophage markers such as Arg1 and CD206 remained unchanged after AGEs stimulation. AGEs significantly increased RAGE expression in macrophages and activated NF-*κ*B pathway, and the aforementioned effects were partly abolished by administration of anti-RAGE antibody or NF-*κ*B inhibitor PDTC. In conclusion, our results suggest that AGEs enhance macrophage differentiation into proinflammatory M1 phenotype at least partly via RAGE/NF-*κ*B pathway activation.

## 1. Introduction

Diabetes mellitus (DM) increases the risk of cardiovascular disease by 2–4-fold through accelerated atherosclerosis [1]. However, the exact mechanisms underlying accelerated atherosclerosis in DM patients are not fully understood.

Monocytes and macrophages are key players in the initiation and progression of atherosclerosis [2, 3]. Macrophages exhibit functional heterogeneity and plasticity, grossly differentiating into classically activated macrophages (M1) upon induction by lipopolysaccharides (LPS) and interferon-*γ* (IFN-*γ*) and alternatively activated macrophages (M2) upon induction by interleukin-4 (IL-4)/interleukin-13 (IL-13) or anti-inflammatory cytokines, such as interleukin-10 (IL-10) or transforming growth factor-*β* (TGF-*β*) [4, 5]. M1 polarized macrophages are characterized by upregulated expression of CD11c and CD86 and expression of several proinflammatory mediators, such as inducible nitric oxide synthase (iNOS), tumor necrosis factor-*α* (TNF-*α*), interleukin-1*β* (IL-1*β*), and interleukin-6 (IL-6), while M2 polarized macrophages are characterized by CD163, CD206, and Arginase 1 (Arg1) expression and secretion of anti-inflammatory cytokines including TGF-*β* and IL-10 [6–8]. There is evidence that atherosclerotic lesions contain both M1 and M2 macrophages: M1 macrophages play key roles in plaque progression and contribute to an inflammatory state [9], while M2 macrophages predominate in the plaques of many atherosclerosis regression models and contribute to inflammation resolution [10, 11]. Overall, M1/M2 ratio is a major determinant of plaque development [12].

Advanced glycation end products (AGEs), a heterogeneous group of molecules formed by irreversible nonenzymatic reactions between proteins and sugar residues in a hyperglycemic milieu, augment cardiovascular risk in patients with diabetes by stimulating inflammation and promoting atheroma formation [13]. In addition to macrophages, the crucial role of AGEs in the pathogenesis of atherogenesis has been established [14]; however, the interaction between these two players has not been fully explored. The present study therefore tests the hypothesis that AGEs enhance macrophage polarization into the M1 phenotype as a potential key mechanism underlying the increased inflammation and accelerated atherosclerosis observed in DM patients.

## 2. Materials and Methods

### 2.1. Mice

Male C57BL/6 mice (8 to 10 weeks old) were purchased from Slac Laboratory Animal Co., Ltd. (Shanghai, China) and housed under specific pathogen-free conditions. All animal experiments were approved by the Ethics Committee of Xinhua Hospital affiliated to Shanghai Jiaotong University School of Medicine.

### 2.2. Preparation and Culture of Bone Marrow Derived Macrophages

The mice were anesthetized by using pentobarbital (50 mg/kg, i.p.) to minimize suffering and killed by cervical dislocation without recovery from anesthesia. Tibias and femurs then were removed under sterile conditions. The bone ends were cut and marrow was flushed using PBS supplemented with 2% Fetal Bovine Serum (FBS, Gibco, Australia, Cat. number 10099-141). A single cell suspension was obtained by filtering the cells through a 40 *μ*m strainer (BD Falcon). Then the suspension was centrifuged at 1500 rpm for 5 min and the cell pellet was resuspended in culture medium. Bone marrow cells were cultured for 7 days in Dulbecco's modified Eagle's medium (DMEM) High Glucose (HyClone, Beijing, China, Cat. number SH30022.01B) supplemented with 10% FBS, 2 mM L-glutamine (Sigma-Aldrich, Missouri, USA, Cat. number 59202C), 100 U/mL penicillin and 100 *μ*g/mL streptomycin (Beyotime, Jiangsu, China, Cat. number C0222), and 50 ng/mL M-CSF (PeProtech, New Jersey, USA, Cat. number 315-02). Cells were harvested on day 7 for further experiments. Macrophages (>95%) were identified by flow cytometry with anti-CD11b-APC and anti-F4/80-FITC (eBioscience, California, USA, Cat. numbers 17-0112 and 11-4801) staining.

### 2.3. Cell Viability Assay

For cell viability detection, a CCK-8 assay was performed. Bone marrow derived macrophage (BMDM) cells (5 × 10^4^/well) were seeded in 96-well plates, cultured overnight, and then incubated with different concentrations (20, 100, 250, and 500 *μ*g/mL) of AGE-BSA (Anyan-bio Technology, Shanghai, China, Cat. number AY-4710P). BSA (500 *μ*g/mL, Sigma-Aldrich, Missouri, USA, Cat. number A1933) was used as control vehicle. Each concentration group was tested in triplicate in three replicate wells. After incubation for 24 h at 37°C with 5% CO_2_, 10 *μ*L CCK-8 solution (DOJINDO, Kumamoto, Japan, Cat. number CK-04) was added to each well and incubated for another 4 h. The optical density (OD) values were measured at 450 nm using a microplate reader (BioTek, USA). The cell viability rate of the treated cells was calculated as relative values to the control group.

### 2.4. Real Time PCR Analysis

Total RNA was extracted from the cells using TRIzol reagent (Takara, Liaoning, China, Cat. number 9109) according to the manufacturer's instructions, and 1 *μ*g of total RNA was reverse transcribed to cDNA using the PrimeScript RT Master Mix kit (Takara, Liaoning, China, Cat. number RR036A) following the manufacturer's instructions. Real time PCR array analysis was performed by using the SYBR Premix Ex TaqTM kit (Takara, Liaoning, China, Cat. number RR420A) in a total volume of 20 *μ*L with 2 *μ*L of cDNA primers (0.2 mM each), 10 *μ*L of SYBR Green, and 0.4 *μ*L Rox Dye II. The standard PCR conditions consisted of 95°C for 30 sec, followed by 40 cycles of 95°C for 5 sec and 60°C for 34 sec, with a final dissociation stage, and the samples were run on an ABI 7500 detector (Applied Biosystems, California, USA). The amounts of target genes were determined and normalized to the amount of GAPDH cDNA (Sangon Biotech, Shanghai, China, Cat. number B661304). The sequences of the primers (Sangon Biotech, Shanghai, China) for the target genes are as follows: Fwd 5′-TCTGCAAGAGACTTCCATCCA-3′ and Rev 5′-AGTCTCCTCTCCGGACTTGT-3′ for IL-6; Fwd 5′-GGTGCCTATGTCTCAGCCTC-3′ and Rev 5′-CCACTTGGTGGTTTGTGAGTG-3′ for TNF-*α*; Fwd 5′-TGCACTACCAAAGCCACAAG-3′ and Rev 5′-TGATCCTCATGCCAGTCAGT-3′ for IL-10; Fwd 5′-AATCTTGGAGCGAGTTGTGG-3′ and Rev 5′-GCAGCCTCTTGTCTTTGACC-3′ for iNOS; Fwd 5′-GCCTTTGTTGATGTCCCTAAT-3′ and Rev 5′-GCACCACACTGACTCTTCCA-3′ for Arg1; Fwd 5′-ACGCAGAAGGACATCAAACC-3′ and Rev 5′-TCTCCAAGAGGACGACTGG-3′ for RAGE.

### 2.5. Cytokines Detected by Enzyme-Linked Immunospecific Assays (ELISA)

After adjusting a single cell suspension of BMDM to a density of 1 × 10^6^ cells/mL, 1 mL cell suspension was added into each well of 12-well plates. BMDM cells were then treated with different concentrations of AGEs (20, 100, and 500 *μ*g/mL) on the next day. The cells incubated with 500 *μ*g/mL BSA were as control groups. After another 24 h incubation, the supernatant fluid was collected and levels of TNF-*α*, IL-6, and IL-10 were determined by commercially available ELISA kits (Neobioscience, Guangdong, China) according to the manufacturer's instructions.

### 2.6. Flow Cytometry (FCM) Analysis

Antibodies such as anti-iNOS-Alexa Fluor 488 (Cat. number 53-5920), anti-CD86-PE (Cat. number 12-0862), and anti-CD11c-PE (Cat. number 12-0114) were purchased from eBioscience (California, USA). Anti-CD206-FITC antibody was purchased from Biolegend (California, USA, Cat. number 141703). Anti-Arg1-PE antibody was purchased from R&D systems (Minnesota, USA, Cat. number IC5868P). For pathway research, FCM was used as previously described [15, 16]. Anti-phospho-P65 antibody (Cat. number 3033) and anti-P65 antibody (Cat. number 8242) were purchased from Cell Signaling Technology (Massachusetts, USA). The primary antibody against RAGE for FCM was purchased from Abcam (Cambridge, UK, Cat. number ab3611). The secondary antibody conjugated with Alexa Fluor 488 was purchased from Beyotime (Jiangsu, China, Cat. number A0423). The antibody for blocking and neutralizing RAGE was purchased from R&D systems (Minnesota, USA, Cat. number AF1179). The NF-*κ*B inhibitor ammonium pyrrolidinedithiocarbamate (PDTC) was purchased from Abcam (Cambridge, UK, Cat. number ab141406). Results were acquired by a BD Canto II flow cytometer (BD Biosciences, USA) and analyzed by the FlowJo software (Tree Star, USA).

### 2.7. Statistical Analysis

All the results were expressed as mean ± SD. Student's *t*-test was used for comparison between two groups. One-way analysis of variance (ANOVA) was used to assess the differences between multiple groups, and post hoc testing was completed by Fisher's least significant differences (LSD). In addition, the difference in NF-*κ*B activation triggered by AGEs with or without anti-RAGE antibody pretreatment was examined by a two-way ANOVA analysis with absence/presence of anti-RAGE antibody and time points as factors. Analysis was performed by SPSS software 19.0 (SPSS Inc., Chicago, USA) for Windows. A value of *p* < 0.05 was considered statistically significant.

## 3. Results

### 3.1. Effects of AGEs on Cell Viability

**Figure 1:**
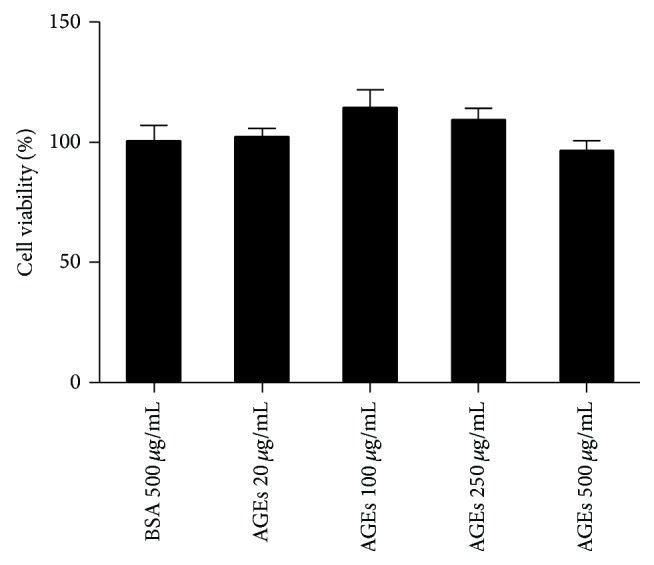
Viability analysis of BMDM cells after treatment with AGEs. Cells (5 × 10^4^/well) were treated with AGEs (20–500 *μ*g/mL) for 24 h or with 500 *μ*g/mL BSA as controls. Cell viability was determined by CCK-8 assay. Compared with BSA, AGEs (20–500 *μ*g/mL) did not affect macrophages viability. Each bar represents the mean value ± SD of triplicate determinations, representative of three independent experiments.

BMDM cells were incubated with different concentrations of AGEs (20, 100, 250, and 500 *μ*g/mL) for 24 h. Cells incubated with 500 *μ*g/mL BSA were used as control groups. In CCK-8 assays, AGEs (20–500 *μ*g/mL) did not affect cell viability at 24 h (Figure 1). Based on these results, concentrations of AGEs among the 20–500 *μ*g/mL range were chosen for subsequent experiments.

### 3.2. Effects of AGEs on Macrophage Production of IL-6, TNF-*α*, and IL-10

**Figure 2:**
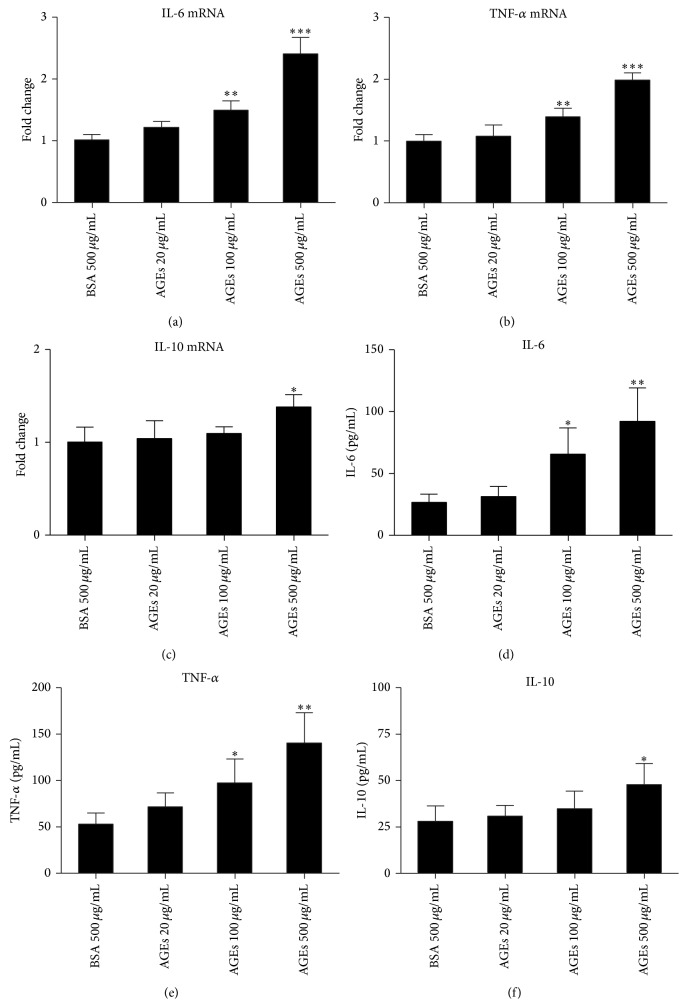
Effects of AGEs on production of IL-6, TNF-*α*, and IL-10. Cells were cultured with AGEs for 24 h, using BSA 500 *μ*g/mL as controls. The mRNA levels were detected by real time PCR. Secretion of cytokines was detected by ELISA. AGEs upregulated mRNA expression of IL-6 (a) and TNF-*α* (b) in a dose dependent manner. Highest concentration of AGEs also increased mRNA expression of IL-10 (c). AGEs significantly increased secretion of IL-6 (d) and TNF-*α* (e). Highest concentration of AGEs also increased IL-10 secretion (f). ^*∗*^ *p* < 0.05; ^*∗∗*^ *p* < 0.01; and ^*∗∗∗*^ *p* < 0.001 compared with control. Each bar represents the mean value ± SD of triplicate determinations, representative of three independent experiments.

M1 macrophages usually predominantly produce proinflammatory cytokines such as IL-6 and TNF-*α*, while M2 macrophages always secrete more anti-inflammatory cytokines such as IL-10. Thus mRNA levels of proinflammatory cytokines (IL-6, TNF-*α*) and anti-inflammatory cytokine (IL-10) in macrophages were detected after incubation with different concentrations of AGEs (20, 100, and 500 *μ*g/mL) or BSA (500 *μ*g/mL) for 24 h. AGEs significantly increased the mRNA expression of IL-6 and TNF-*α* in a dose dependent manner (Figures 2(a) and 2(b)), while mRNA level of IL-10 was slightly increased only under stimulation of the highest AGEs concentration (Figure 2(c)). Effects of AGEs on the secretion of above cytokines were determined in supernatant fluids by ELISA. AGEs still significantly increased IL-6 and TNF-*α* protein levels in a dose dependent manner (Figures 2(d) and 2(e)), and only the highest concentration of AGEs induced IL-10 secretion (Figure 2(f)). These data demonstrate that AGEs predominantly induce macrophages to secrete proinflammatory cytokines.

### 3.3. Effects of AGEs on the Expression of iNOS and Arg1 in Macrophages

**Figure 3:**
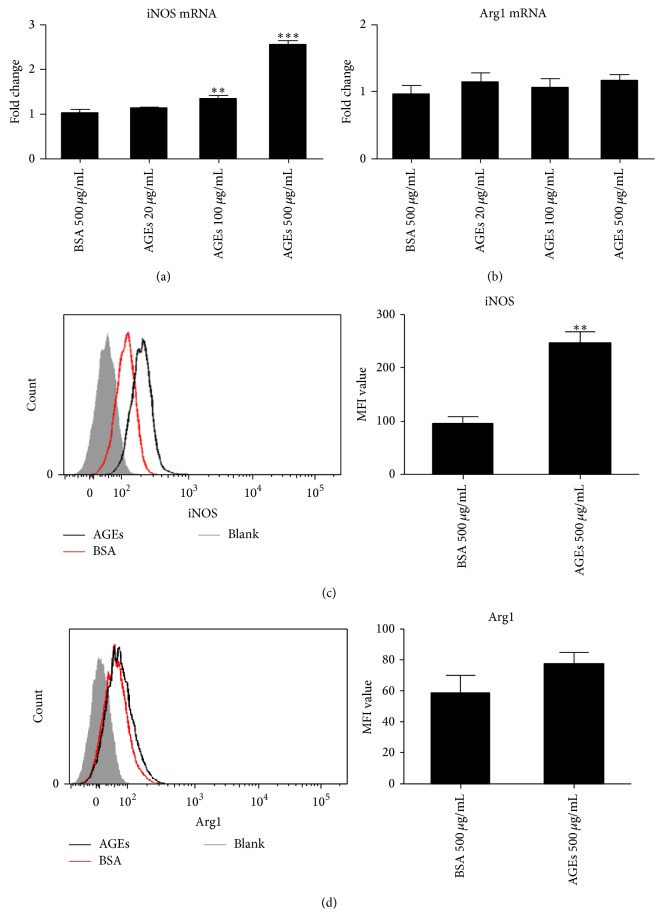
Effects of AGEs on mRNA levels and expressions of iNOS and Arg1. For mRNA evaluation, the cells were cultured with different concentrations of AGEs for 24 h. For protein expression detection, the cells were incubated with AGEs 500 *μ*g/mL for 24 h. BSA 500 *μ*g/mL was used as a control. The mRNA levels were assayed by real time PCR, and protein expression was measured by FCM. AGEs significantly upregulated iNOS mRNA expression in a dose dependent manner (a) but did not affect Arg1 mRNA level (b). AGEs increased iNOS expression (c) but did not affect the expression of Arg1 (d). ^*∗*^ *p* < 0.05; ^*∗∗*^ *p* < 0.01; and ^*∗∗∗*^ *p* < 0.001 compared with control. MFI: mean fluorescence intensity. Each bar represents the mean value ± SD of triplicate determinations, representative of three independent experiments.

It is widely accepted that iNOS is a marker for M1 while Arg1 is a marker for M2 macrophages [7]. To elucidate the effects of AGEs on iNOS and Arg1 expression in BMDM, mRNA levels of iNOS or Arg1 in AGEs stimulated macrophages were assessed by real time PCR. 24 hours after stimulation, mRNA level of iNOS was dose dependently upregulated while Arg1 mRNA expression remained unchanged (Figures 3(a) and 3(b)). Expressions of iNOS and Arg1 in macrophages incubated with AGEs 500 *μ*g/mL for 24 h were determined by FCM. Similarly, AGEs still significantly upregulated iNOS production without affecting Arg1 expression (Figures 3(c) and 3(d)).

### 3.4. Effects of AGEs on the Expression of Surface Markers on Macrophages

**Figure 4:**
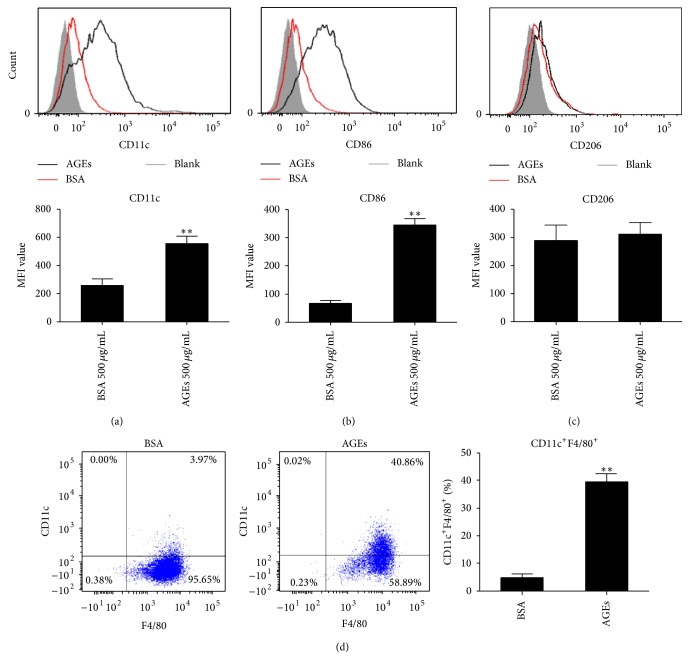
Effect of AGEs on expressions of surface markers. Cells were cultured with AGEs or BSA 500 *μ*g/mL for 24 h. The markers were measured by FCM. AGEs increased CD11c (a) and CD86 (b) expression but did not affect CD206 expression (c). Double staining with CD11c and F4/80 indicated that AGEs increased F4/80^+^ CD11c^+^ double positive macrophages (d). ^*∗*^ *p* < 0.05, and ^*∗∗*^ *p* < 0.01 compared with control. MFI: mean fluorescence intensity. Each bar represents the mean value ± SD of triplicate determinations, representative of three independent experiments.

M1 macrophages usually express a higher level of cell surface markers such as CD11c and CD86, while M2 macrophages express a higher level of mannose receptor (CD206) [6, 7]. We thus evaluated the impacts of AGEs on the expressions of these surface markers by FCM. As expected, expressions of CD11c and CD86 were significantly upregulated on macrophages after AGEs stimulation (Figures 4(a) and 4(b)). Nevertheless, the level of CD206 remained unchanged (Figure 4(c)). Double staining with F4/80 and CD11c further indicated that AGEs increased CD11c expression on F4/80^+^ macrophages (Figure 4(d)).

### 3.5. Role of RAGE/NF-*κ*B Pathway on Macrophage Polarization after AGEs Stimulation

**Figure 5:**
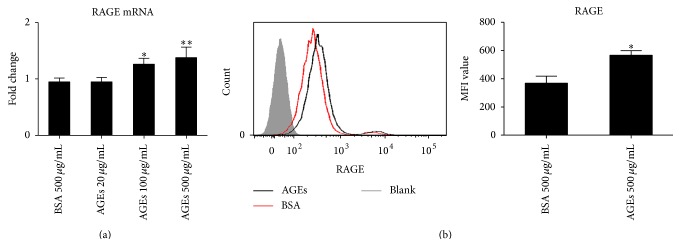
Role of AGEs on the expression of RAGE. For mRNA evaluation, the cells were cultured with different concentrations of AGEs for 24 h. For protein expression detection, the cells were incubated with AGEs 500 *μ*g/mL for 24 h. BSA 500 *μ*g/mL was used as a control. The mRNA levels were assayed by real time PCR and protein expression was assayed by FCM. AGEs upregulated RAGE mRNA expression in a dose dependent manner (a). AGEs significantly increased RAGE protein expression (b). ^*∗*^ *p* < 0.05, and ^*∗∗*^ *p* < 0.01 compared with control. MFI: mean fluorescence intensity. Each bar represents the mean value ± SD of triplicate determinations, representative of three independent experiments.

RAGE is the most important and common receptor which interacts with AGEs. What is more, AGEs/RAGE/NF-*κ*B axis plays critical roles in inducing various kinds of cells to inflammatory conditions [17, 18]. Thus we hypothesized that the axis might be involved in AGEs induced macrophage polarization. To confirm the hypothesis, firstly we assayed the effect of AGEs on expression of RAGE by real time PCR and FCM. As a result, AGEs treatment significantly upregulated not only mRNA level but also protein level of RAGE expression in macrophages (Figures 5(a) and 5(b)).

**Figure 6:**
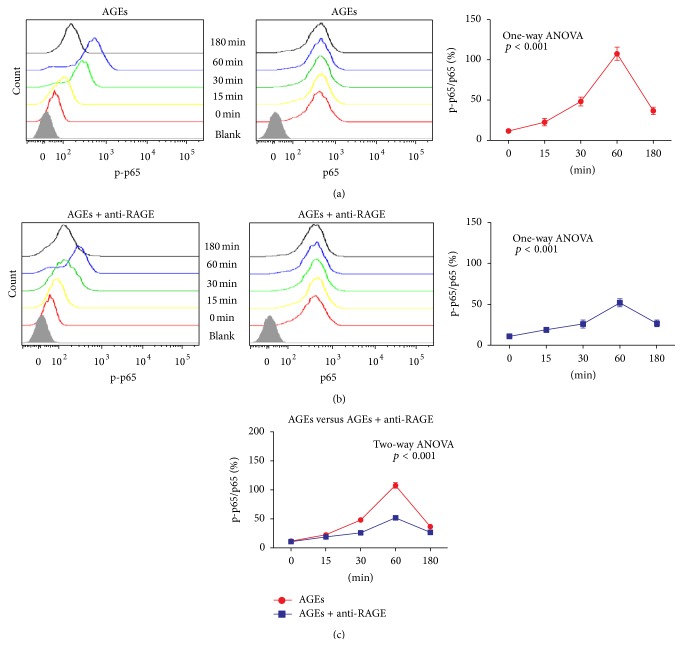
Role of AGEs on RAGE/NF-*κ*B pathway. The cells were incubated with AGEs 500 *μ*g/mL for different time. The p-p65 and total p65 protein were measured as MFI (mean fluorescence intensity) by FCM. The NF-*κ*B pathway was activated by AGEs (a). After pretreatment with anti-RAGE antibody for 60 min, NF-*κ*B was still activated by AGEs (b). NF-*κ*B pathway activation was partly inhibited by anti-RAGE antibody (c). Each point represents the mean value ± SD of triplicate determinations, representative of three independent experiments.

Next, we evaluated the effect of AGEs on activation of NF-*κ*B pathway in macrophages. Because activation of NF-*κ*B pathway is associated with phosphorylation of NF-*κ*B, phospho-NF-*κ*B-p65 (p-p65) and NF-*κ*B-p65 (p65) were measured as mean fluorescence intensity (MFI) by FCM in different time points after AGEs stimulation. The p-p65 level increased within 15 min after treatment with AGEs. Moreover, the effect peaked at 60 min and decreased thereafter. In contrast, the total NF-*κ*B-p65 (p65) level remained unchanged over the time after AGEs treatment. Furthermore, the p-p65/p65 ratio increased significantly shortly after AGEs stimulation, peaked at 60 min, and then decreased (Figure 6(a)). These data indicate that AGEs can activate the NF-*κ*B pathway.

To further identify the role of AGEs/RAGE axis on activation of NF-*κ*B, RAGE neutralizing antibody was used to block RAGE receptors. BMDM cells were pretreated with anti-RAGE antibody (50 *μ*g/mL) for 60 min; then AGEs were added into the culture medium to stimulate the cells. FCM results indicated that p-p65 still increased within 15 min and peaked at 60 min and decreased thereafter, while total p65 levels remained unchanged after AGEs treatment. The p-p65/p65 ratio changed in the same way as p-p65 level (Figure 6(b)). The results demonstrate that AGEs can still activate the NF-*κ*B pathway even with RAGE receptor blockade. Of note, the peak of p-p65/p65 ratio was significantly lower and the changes of p-p65/p65 ratios were somehow less in the RAGE blocking group than in the group without anti-RAGE antibody pretreatment (Figure 6(c)). These data show that anti-RAGE antibody can partly inhibit the activation of NF-*κ*B which can be activated by AGEs.

The experiments above demonstrate that AGEs can induce NF-*κ*B activation at least partly through RAGE signaling. Furthermore, the inability of RAGE blocking pretreatment to completely inhibit the NF-*κ*B activation suggests AGEs might interact with other receptors to initiate NF-*κ*B activation.

**Figure 7:**
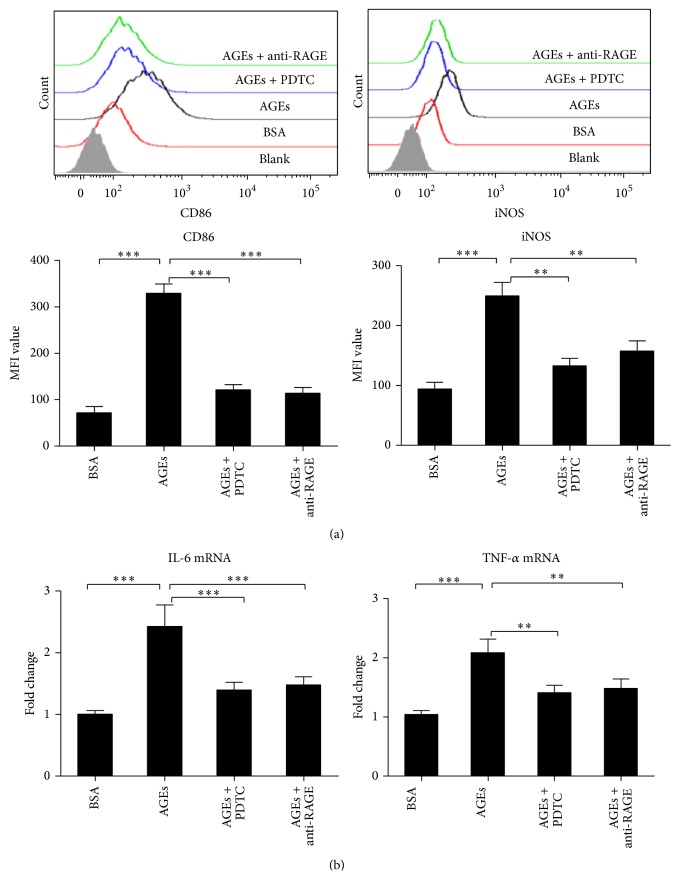
Effects of RAGE/NF-*κ*B pathway blocking treatment on M1 markers expression induced by AGEs. The cells were cultured with AGEs or BSA 500 *μ*g/mL for 24 h. For pathway blocking treatment, cells were pretreated with anti-RAGE antibody (50 *μ*g/mL) or PDTC (100 *μ*M) for 60 min before AGEs stimulation. The mRNA levels were assayed by real time PCR and protein expression was assayed by FCM. Pretreatment with anti-RAGE Ab or PDTC attenuated effects of AGEs on expression of iNOS and CD86 (a). Pretreatment with anti-RAGE Ab or PDTC attenuated effects of AGEs on the expression of mRNA of IL-6 and TNF-*α* (b). ^*∗*^ *p* < 0.05; ^*∗∗*^ *p* < 0.01; and ^*∗∗∗*^ *p* < 0.001 compared with AGEs group. MFI: mean fluorescence intensity. Each bar represents the mean value ± SD of triplicate determinations, representative of three independent experiments.

The role of RAGE/NF-*κ*B pathway on M1 markers after AGEs stimulation was then investigated. Anti-RAGE antibody and a specific inhibitor of NF-*κ*B (PDTC) were used to block the RAGE/NF-*κ*B pathway, respectively. The expressions of M1 markers including iNOS and CD86 were evaluated by FCM, and the mRNA levels of IL-6 and TNF-*α* were evaluated by real time PCR. As shown in Figure 7(a), AGEs significantly upregulated iNOS and CD86 expression and these effects were significantly attenuated by blocking RAGE with anti-RAGE antibody. Moreover, PDTC also significantly inhibited AGEs induced iNOS and CD86 expression.  Figure 7(b) shows that AGEs induced expressions of the proinflammatory cytokines (IL-6 and TNF-*α*) were significantly inhibited by anti-RAGE antibody or PDTC pretreatment, respectively. These results suggest AGEs induce macrophage to express proinflammatory cytokines and M1 markers via RAGE/NF-*κ*B pathway.

## 4. Discussion

In response to hyperglycemia in patients with diabetes, various metabolic mechanisms contribute to the pathogenesis of diabetic complications. The close participation of AGEs in vascular complications has been well documented. In the present study, we find that AGEs not only predominantly induce macrophages to secrete inflammatory cytokines but also induce M1 polarization as evidenced by the iNOS and expressions of surface markers on macrophages. Moreover, AGEs stimulation activates RAGE/NF-*κ*B pathway and blockade of RAGE or NF-*κ*B can attenuate the AGEs effects on macrophage polarization. Taken together, our results indicate that AGEs can induce macrophages polarization into M1 phenotype partly through RAGE/NF-*κ*B pathway and this might be one of the mechanisms responsible for the diabetic vascular complications.

Advanced glycation end products play an important role in the pathogenesis of diabetic complications by inducing inflammation, especially in atherosclerosis, while macrophages and their polarization status dominate the process of atheroma formation and progression. Therefore, investigating the interaction between AGEs and the polarization of macrophages might further reveal the underlying mechanism of diabetic accelerated atherosclerosis. Previous studies have shown that AGEs not only promoted macrophages to secret proinflammatory cytokines [19, 20] but also enhanced their migration ability [21], while Choi et al. recently proved that AGEs enhanced iNOS expression in macrophages [22]. In the present study, we also found AGEs upregulated iNOS production. As to the cytokines expressions, although our results showed the highest concentration of AGEs slightly increased IL-10 production, the expressions of proinflammatory cytokines such as IL-6 and TNF-*α* increased more sharply and dominated the macrophage cytokine expression profile. Thus, our results are in line with previous studies which indicate AGEs induce macrophages into proinflammatory status by enhancement on proinflammatory molecules production. As M1 macrophages are characterized by “proinflammatory” function, the above mentioned data suggest AGEs might prime macrophages into M1 phenotype. However, it is essential to conduct the surface marker study to confirm the macrophages polarization. Unfortunately, no previous studies have evaluated the impact of AGEs on macrophage surface markers alteration. We thus carried out related experiments to elucidate the surface markers shift on macrophages to further ascertain the polarization changes induced by AGEs. As a result, we found the expressions of M1 surface markers including CD11c and CD86 were upregulated, while M2 surface marker CD206 remained unchanged after AGEs treatment. What is more, double staining with CD11c and F4/80, a macrophage-restricted surface marker [23], further suggested that AGEs induce macrophage polarization into M1 phenotype. Taken together, AGEs increased proinflammatory mediators and M1 surface markers on macrophages; these data indicate that AGEs prime macrophage into M1 phenotype.

The underlying mechanism of AGEs mediated macrophage M1 polarization remains largely unknown now. It is known that receptor for AGEs (RAGE) is a multiligand receptor expressed on many cells and mediates the most biological effects of AGEs [24]. Previous study showed that AGEs-RAGE interaction could enhance macrophages migration [21] and promote proinflammatory mediators production such as IL-1*β*, IL-6, and TNF-*α* [25]. However, the role of AGEs-RAGE axis on macrophage polarization was not reported. Consistent with previous studies, we found AGEs-RAGE interaction participated in the signal transduction to increase expressions of IL-6 and TNF-*α* since anti-RAGE antibody pretreatment could partly abate AGEs induced cytokines production. Furthermore, we also found AGEs increased M1 markers expression via RAGE signaling since pretreatment with anti-RAGE antibody attenuated M1 markers expression. Thus, our data prove that AGEs-RAGE interaction plays a pivotal role in AGEs induced M1 polarization. Former research has established that AGEs-RAGE axis could activate several central transcription factors including nuclear factor- (NF-) *κ*B, cAMP-response-element-binding protein- (CREB-) 1, early growth response- (EGR-) 1, and activator protein- (AP-) 1 [26]. Among them, NF-*κ*B is the key target of AGEs/RAGE signaling in atherosclerosis. It could mediate proinflammatory response in various kinds of cells [18, 27], as well as in macrophages [28]. In the present study, we found AGEs activated NF-*κ*B and its activation could be partly inhibited by RAGE blocking pretreatment. That means AGEs can induce NF-*κ*B activation partly via RAGE signaling. It should be noted that NF-*κ*B has been found to participate in several molecules induced macrophage M1 polarization, for example, C reactive protein (CRP) and Maltose-binding protein (MBP) [29, 30]. Thus NF-*κ*B probably will be implicated in AGEs-RAGE axis induced M1 polarization. As we found in this study, administration of anti-RAGE antibody partly suppressed NF-*κ*B activation, and pretreatment with anti-RAGE antibody or PDTC abated effects of AGEs on priming macrophages to the M1 phenotype. These results demonstrate AGEs induce macrophages into the M1 phenotype at least partly through the RAGE/NF-*κ*B pathway.

Of note, in addition to RAGE, AGEs also act as ligands for several other receptors such as AGE-receptor complex (AGE-R1/OST-48, AGE-R2/80K-H, and AGE-R3/galectin-3) and toll-like receptor-4 (TLR-4) [31, 32]. Furthermore, there is evidence that AGEs can induce NF-*κ*B activation through TLR4 signaling [32], and TLR4 signaling can mediate M1 polarization [33, 34]. In the current experiment, we found the RAGE blockade pretreatment could not totally abolish the activation of NF-*κ*B triggered by AGEs. Thus, this phenomenon suggested, besides RAGE, AGEs probably could interact with other receptors to initiate cell signaling and then induced NF-*κ*B activation to mediate macrophage M1 polarization. What is more, previous studies have demonstrated that several other pathways, such as C-Jun N-terminal kinase (JNK), Notch, and JAK/STAT signaling pathway, are involved in macrophage polarization [35]. Thus, further work is warranted to reveal other receptors and pathways underlying AGEs induced macrophage polarization.

As is well known, macrophages respond to an extraordinary diversity of environmental stimuli, including different microbial products and various cytokines. Although M1 and M2 macrophages coexist in the atherosclerotic lesions, a large body of experimental evidence supports the contribution of M1 macrophages in atherosclerosis progression and lesion destabilization [12, 36]. Very recently, Fadini et al. found an increased M1/M2 polarization ratio in prediabetic and diabetic patients [37, 38] suggesting an unfavorable M1/M2 polarization might be a crucial underlying mechanism responsible for accelerated atherosclerosis in diabetic patients. Although hyperglycemia was found to induce macrophages to produce proinflammatory molecules [39, 40], it remains unknown whether it can also affect macrophage polarization. In the present study, we found that AGEs induced macrophages polarization into M1 phenotype. Such findings suggest that AGEs, the main pathogenic factor of diabetes, may be one of the chief culprits to disturb the M1/M2 ratio. By promoting macrophage polarization into M1 phenotype, AGEs might act as a major player linking diabetes and accelerated atherosclerosis.

## 5. Conclusion

The present study suggests that AGEs enhance mouse bone marrow derived macrophage polarization toward a proinflammatory M1 phenotype at least partly via activating the RAGE/NF-*κ*B pathway. These data thus underscore a potential role of AGEs on affecting macrophage polarization. Strategies which might block or even reverse the effect of AGEs on macrophage M1 polarization might be novel ways to alleviating the atherosclerosis progression in diabetic patients.
